# Orthotopic model of lung cancer: isolation of bone micro-metastases after tumor escape from Osimertinib treatment

**DOI:** 10.1186/s12885-021-08205-9

**Published:** 2021-05-10

**Authors:** Ulrich Jarry, Mégane Bostoën, Raphaël Pineau, Laura Chaillot, Valentine Mennessier, Pierre Montagne, Emilie Motte, Marjorie Gournay, Arnaud Le Goff, Thierry Guillaudeux, Rémy Pedeux

**Affiliations:** 1 Université Rennes 1, UMS 3480 CNRS/US018 INSERM BIOSIT, Laboratoire Commun ONCOTRIAL, Rennes, France; 2 Biotrial Pharmacology, Unité De Pharmacologie Préclinique, Rennes, France; 3 INSERM U1242 COSS, Université Rennes 1, Clcc Eugène Marquis, Rennes, France

**Keywords:** Orthotopic lung tumor model, Metastasis, Bioluminescence, EGFR TK inhibitor, Tumor escape, Osimertinib

## Abstract

**Background:**

Osimertinib is a third generation tyrosine kinase inhibitor (TKI) that targets the epidermal growth factor receptor (EGFR) in lung cancer. However, although this molecule is not subject to some of the resistance mechanisms observed in response to first generation TKIs, ultimately, patients relapse because of unknown resistance mechanisms. New relevant non-small cell lung cancer (NSCLC) mice models are therefore required to allow the analysis of these resistance mechanisms and to evaluate the efficacy of new therapeutic strategies.

**Methods:**

Briefly, PC-9 cells, previously modified for luciferase expression, were injected into the tail vein of mice. Tumor implantation and longitudinal growth, almost exclusively localized in the lung, were evaluated by bioluminescence. Once established, the tumor was treated with osimertinib until tumor escape and development of bone metastases.

**Results:**

Micro-metastases were detected by bioluminescence and collected for further analysis.

**Conclusion:**

We describe an orthotopic model of NSCLC protocol that led to lung primary tumor nesting and, after osimertinib treatment, by metastases dissemination, and that allow the isolation of these small osimertinib-resistant micro-metastases. This model provides new biological tools to study tumor progression from the establishment of a lung tumor to the generation of drug-resistant micro-metastases, mimicking the natural course of the disease in human NSCLC patients.

**Supplementary Information:**

The online version contains supplementary material available at 10.1186/s12885-021-08205-9.

## Background

Lung cancer is one of the most common and deadliest forms of cancer worldwide. Non-small cell lung cancer (NSCLC) accounts for nearly 85% of cases [1, 2] and frequently spreads to the bones (30 to 40% of patients) [3, 4]. Despite platinum-based chemotherapy, radiotherapy and/or surgery, the median survival time after diagnosis remains low (≈ 10 months) [5]. Since the early 2000s, several oncogenic factors have been identified for patients with NSCLC, including the epidermal growth factor receptor (EGFR). Point mutations in exon 19 [del 19] and exon 21 (L858R) are present in approximately 15% of Caucasian patients and in 35% of Asian patients [6, 7]. First and second generation EGFR inhibitors have shown encouraging results [8] and they are currently used as a first line treatment for patients with NSCLC carrying EGFR mutations [9]. Unfortunately, resistance mechanisms frequently occur after 9 to 13 months of treatment [10, 11], especially the appearance of the T790M mutation [12, 13]. Osimertinib is a third generation EGFR inhibitor, effective even against tumors bearing the T790M mutation. It has been clinically demonstrated to elicit strong and long-lasting responses [14]. Despite its efficacy, other resistance mechanisms also occur in response to osimertinib (e.g. EGFR G796 / C797, L792 and L718 / G719 mutations, activation of downstream oncogenes, such as MET, KRAS and PIK3CA) [15, 16]. In this context, developing a relevant NSCLC model allowing i) the analysis of these resistance mechanisms and ii) the evaluation of new therapeutic strategies that bypass these resistance mechanisms represents an essential tool for medical research.

Herein, we describe an orthotopic model of NSCLC protocol that led to lung primary tumor nesting and, after osimertinib treatment, by metastases dissemination, observed mostly within the bones. Using this model we are able to isolate the bone micro-metastases that appear at the beginning of tumor escape following osimertinib treatment.

Briefly, the human NSCLC cell line PC-9, previously transfected in order to express luciferase, was injected into the tail vein of immunodeficient NSG mice (*NOD.Cg-Prkdcscid Il2rgtm1Wjl/SzJ*) in order to establish a relevant orthotopic and bioluminescent NSCLC model in mice. Tumor implantation and longitudinal growth were monitored by bioluminescence. When tumors were established, the mice were treated daily with osimertinib until tumor escape. Metastases, mostly localized in the bones, were then collected for further analysis.

## Methods

### Preparation of bioluminescent human NSCLC cell line

1–1: The PC-9 cell line (formerly known as PC-14; ACACC90071810; Sigma-Aldrich, St. Louis, MO) was cultured in RPMI (Dutscher, Brumath, France) supplemented with 10% heat-inactivated fetal bovine serum (FBS, Dutscher) and 2 mM L-glutamine (Dutscher). The A549 cell line (CCL-185™, ATCC, Manassas, VA) was cultured in low-glucose DMEM (Dutscher) supplemented with 10% heat-inactivated FBS and 2 mM L-glutamine. Cells were cultured using routine cell culture techniques. Of note, while A549 cells do not carry mutations in the *EGFR* gene and are not sensitive to osimertinib, PC-9 cells carry a Glu746-Ala750 deletion mutation in exon 19 of the *EGFR* gene [17] and are sensitive to osimertinib [18].

1–2: For luciferase expression, PC-9 cells were transfected with the pGL4.51 [luc2/CMV/NEO] vector (Promega, Madison, WI) and A549 cells were transduced with RediFect Red-Fluc-Puromycin lentiviral particles (PerkinElmer, Waltham, MA) according to the manufacturer’s instructions. PC-9 and A549 Luc-positive (Luc^+^) cells were maintained under selection with G418 (Dutscher) and puromycin (Sigma-Aldrich), respectively.

1–3: Prior to injection, NSCLC Luc^+^ cells (70–80% confluence) were harvested. Briefly, cells were washed with phosphate-buffered saline (PBS) and detached using 0.25% trypsin-EDTA. Trypsin was neutralized with medium containing 10% FBS. After centrifugation (350 x g for 5 min), cells were resuspended in PBS.

### Mice

2- : The following procedure involving animals was performed according to institutional guidelines (Agreement APAFIS # 8887; regional ethics committee of Brittany; France). Balb/c nude (BALB/cAnNRj-Foxn1nu/nu) and Nod-Scid (NOD.CB17-Prkdcscid/Rj) mice were purchased from Janvier Labs (Saint Berthevin, France). NSG (*NOD.Cg-Prkdcscid Il2rgtm1Wjl/SzJ)* mice were purchased from Charles River Laboratories (Wilmington, MA). Mice were bred in the animal facility of the University of Rennes 1 (Arche, UMS Biosit, Rennes, France) under specific pathogen-free (SPF) environment and used in the experiments at 6–8 weeks of age. Vendor health reports indicated that the mice were free of known all viral, bacterial and parasitic pathogens listed in the Federation of European Laboratory Animal Science association's (FELASA) recommendations upon arrival at the facility. The mice were acclimatized to the environmental conditions at least 7 days before use. The animals were housed with a 12-h day-night cycle with lights on at 8:00 pm in a room with controlled temperature (22 ± 1 °C), with free access to food and water in filter top cages (Tecniplast, France) enriched with a mouse house (3–5 mice per cage). The animals’ health status was monitored throughout the experiments by a health surveillance program in accordance with the FELASA guidelines. All in vivo experiments are recapitulated in Supp Table 1. With regards to subcutaneous (sc) tumor model development, tumorigenicity was firstly assessed by using 10 Balb/c nude mice. The effects of osimertinib and paclitaxel were evaluated using 18 Balb/c nude mice, each implanted with PC-9 Luc^+^ cells: 6 untreated, 6 treated with paclitaxel and 6 treated with osimertinib. With regards to the orthotopic models using A549 Luc^+^ cells, 6 Balb/c nude mice were used for intercostal implantation and 17 Nod-Scid mice were used for intratracheal implantation of cells. For PC9 and A549 Luc^+^ cells iv implantation, assays were performed prior to the beginning of the experiments using 10 Balb/c nude mice, 10 Nod-Scid mice and 10 NSG mice. Seven [7] mice were injected iv with PC-9 Luc^+^ cells, treated with osimertinib and used for subsequent metastasis isolation. Tumor growth was assessed by bioluminescence and, for the sc model, also measured with calipers.

### Subcutaneous implantation

3–1: NSCLC Luc^+^ cells were carefully resuspended by pipetting before being drawn into a syringe with a 25 G needle (1 × 10^6^ cells in 50 μL / mouse).

3–2: The mouse was anesthetized using an isoflurane chamber prior to dorsal subcutaneous (sc) injection.

### Tail vein injection

4–1: NSCLC Luc^+^ cells were carefully resuspended by pipetting before being drawn into a syringe with a 25 G needle (1 × 10^6^ cells in 200 μL / mouse).

4–2: The mouse was placed under a beaker and the tail was firmly held with the experimenter’s non-dominant hand.

4–3: The tail was cleaned using alcohol pad to allow a better visualization of the tail vein.

*Note: Heat lamps and heated surgery pads may also be used to induce tail vein dilatation.*

4–4: The mouse tail was extended and placed parallel to the table.

4–5: The needle was inserted into the tail vein from the distal end and the needle was held steady.

4–6: The cell suspension was injected by gently pushing on the syringe’s plunger. The suspension had to flow unimpeded into the vein. If not, this step was repeated in a more proximal location on the tail.

4–7: The needle was removed and bleeding was stopped by holding some gauze at the injection site for 20–30 s.

### Intercostal implantation

5–1: NSCLC Luc^+^ cells were carefully resuspended by pipetting before being drawn into a syringe with a 25 G needle (1 × 10^6^ cells in 50 μL / mouse).

5–2: The mouse was anesthetized using an isoflurane chamber and then positioned in a right lateral decubitus position with his nose in an isoflurane nosecone to maintain anaesthesia.

5–3: The mouse was shaved and the injection site was identified using a marker (between the fifth and sixth rib bones and on the right anterior axillary line).

5–4: The cell suspension was quickly injected at a depth of approximately 5 mm.

5–5: The mouse was replaced back in a cage and observed until complete recovery.

### Intratracheal implantation

6–1: NSCLC Luc^+^ cells were carefully resuspended by pipetting before being drawn into a MicroSprayer® Aerosolizer type syringe with a 26 G needle (2.5 × 10^5^ cells in 25 μL / mouse).

6–2: The mouse was anesthetized using an isoflurane chamber and then positioned and placed on his back on a platform, with a bar placed in between their top and bottom incisors to keep their head titled back in order to clearly visualize the throat. The nose of the mouse was kept in an isoflurane nosecone to maintain anesthesia.

6–3: Using a laryngoscope, the cell suspension was slowly injected.

6–4: The mouse was placed back in his cage and observed until complete recovery.

### Bioluminescent tumor monitoring

7–1: Sterile-filtered K^+^ D-luciferin was prepared as described by the manufacturer. For the experiments, we used D-luciferin potassium salt from Interchim (Montluçon, France) at 15 mg/mL.

7–2: The mouse was injected intraperitoneally (ip) with luciferin (7.5 μL/g of body weight).

7–3: The mouse was anesthetized using an isoflurane chamber and then positioned inside the imager (either ventral side down for the sc model or ventral side up for the orthotopic models), with his nose in an isoflurane nosecone to maintain anaesthesia. For these experiments, we used the PhotonIMAGER™ from Biospace Lab (Nesles la Vallée, France), equipped with a highly sensitive cooled CCD camera.

7–4: After image acquisition, routinely performed for 1 min, mouse was placed back in his cage.

*Note: The time profile of the signal acquisition ensures that the signal is acquired while the luciferin is in the saturation state.*

7–5: The data were analyzed with M3 Vision™ software provided by Biospace, using cpm (count per minutes) per cm^2^ and focused on the whole body of the mice.

### Mouse treatments

8- : For the sc model, from day 15 after tumor implantation until the end of the experiment, PC-9 Luc^+^ tumor-bearing mice were treated with osimertinib (Biorbyt Ltd., Cambridge, UK) at 1 mg/kg 5 days per week, or with paclitaxel (Paclitaxel AHCL, Interchim, Monluçon, France) at 20 mg/kg 2 days per week. For the orthotopic models, 3 weeks after injection of PC-9 Luc^+^ cells, the mice were treated with increasing doses of osimertinib from 1 mg/kg to 15 mg/kg by ip injection 5 days per week. This treatment, which first led to tumor signal regression, was administered until tumor escape, which is characterized by an overall increase in the intensity of the luminescence throughout the body of the mice (for at least 2 consecutive acquisitions).

### Tumor micro-metastasis isolation

9–1: K^+^ D-luciferin solution, dissection instruments, culture dishes, 24-well plates, and PC-9 cell culture medium (with added penicillin-streptomycin (Dutscher)) were prepared in sterile conditions.

9–2: PC-9 Luc^+^ tumor-bearing mice were first monitored for bioluminescence as previously described in order to localize metastases.

*Note: The next step should be executed quickly, as the bioluminescence signal decreases rapidly.*

9–3: Immediately after bioluminescence acquisition, the anesthetized mouse was euthanized by cervical dislocation.

9–4: In order to maintain the sterility of the sample, the ventral side of the mouse and the area of the metastases were disinfected with 70% ethanol under the hood of a biological safety cabinet.

9–5: A large incision was made from the throat to the belly, and the rib cage was cut through and removed.

9–6: The lung were removed and placed in a culture dish.

9–7: The skin over the metastatic areas was also incised and, based on the picture from the bioluminescence acquisition, micro-metastases were removed and placed in culture dishes.

9–7: 100 to 200 μL of K^+^ D-luciferin solution was applied to each sample and bioluminescence acquisition was performed.

*Note: Bioluminescence may also be acquired from the whole remaining body of the animal at the end of the procedure in order to verify that lung tumors and micro-metastases were properly removed.*

9–8: Samples from metastases were cut into 4–5 mm^3^ fragments and placed into 24-well plates with 400 μL of media per well. After 4–5 days, when tumor cells had colonized the bottom of the well, the fragments were removed.

*Note: Luciferase expression was checked in the cultured cells.*

### Analysis by immunohistochemistry

10- : Tumor samples were fixed with 4% paraformaldehyde (PFA) in PBS, paraffin-embedded and serially sectioned at a thickness of 4 μm. Sections were stained with hematoxylin and eosin (H&E) or incubated with 2% bovine serum albumin (BSA) prior to immunohistochemistry (IHC) staining for vimentin (clone EPR3776, Abcam, Cambridge, UK). The sections were then incubated with goat anti-rabbit IgG H&L coupled to horseradish peroxidase (HRP; ab205718, Abcam) and immunoreactivity was revealed with a diaminobenzidine (DAB) detection system (Roche Ventana). Slides were scanned using a NanoZoomer 2.0 HT (Hamamatsu Photonics K.K., Hamamatsu, Japan).

### Statistical analysis

Data, expressed as bioluminescence intensity (cpm/cm^2^ or ph/sec/sr) and tumor size (defined as L x l^2^ / 2 in mm^3^), were analyzed using GraphPad Prism 7.0 software (GraphPad Software Inc., San Diego, CA). An unpaired Student’s t test was used to reveal significant differences in tumor growth.

## Results

We describe herein an orthotopic lung cancer tumor model in which tumor growth is assessed by non-invasive bioluminescence. This model of NSCLC allowed us i) to evaluate the efficacy of the 3rd generation tyrosine kinase inhibitor (TKI), osimertinib and ii) upon relapse to detect and extract micro-metastases that could not be detected by macroscopic observation.

**Fig. 1:**
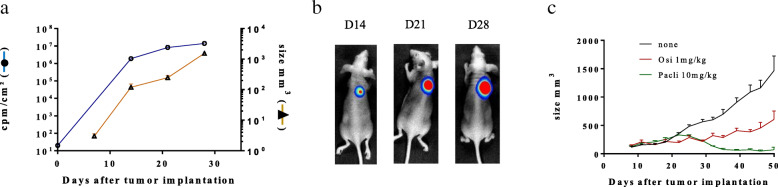
Evaluation of PC9 Luc^+^ cell tumorigenicity and response to osimertinib. PC-9 Luc^+^ cells were injected sc in Balb/c nude mice. Tumor growth was assessed by caliper measurements (**a**) and bioluminescence (**a** & **b**). **a** Tumor sizes are expressed in mm^3^ and tumor bioluminescence intensity is expressed as cpm/cm^2^ for the thoracic area (mean ± SEM, *n* = 5). **b** Pictures show representative results for bioluminescence in tumor-bearing mice on days 14, 21 and 28 after tumor cell injection. **c** Tumor-bearing mice were either treated or not (none) with osimertinib 1 mg/kg, 5 days/week or with paclitaxel (paclitaxel AHCL) 20 mg/kg, 2 days/week, from day 15 after tumor cell injection to the end of the experiment. Tumor growth was assessed by caliper measurements and tumor sizes are expressed in mm^3^ (mean ± SEM, *n* = 6)

Of note, to assess the tumorigenicity of the generated Luc^+^ NSCLC cell line, PC-9 Luc^+^ cells were implanted sc in Balb/c nude mice (Fig. 1). Tumor growth was assessed by both caliper (Fig. 1a) (mm^3^; mean ± SEM, *n* = 5) and bioluminescence (Fig. 1a-b) (cpm/cm^2^; mean ± SEM, n = 5) measurements. As expected, the data show a good correlation between tumor size and bioluminescence intensity. Similar results were obtained using A549 Luc^+^ cells (data not shown). Using the *EGFR*-mutated cell line PC-9 Luc^+^, the efficacy of osimertinib was also evaluated in this sc model in comparison with paclitaxel, used as a standard chemotherapy (Fig. 1c). The results demonstrated that PC9 Luc^+^ cells are sensitive to both treatments in vivo (tumor size comparison at D50, using a Student’s t test (none vs osimertinib: ***, *p* = 0.0002; *n* = 6) (none vs paclitaxel ****, *p* < 0.0001; *n* = 6)). The efficacy of osimertinib was not assessed on tumors derived from A549 Luc^+^ cells, as these cells do not bear an *EGFR* mutation and are thus not sensitive to the drug.

**Fig. 2:**
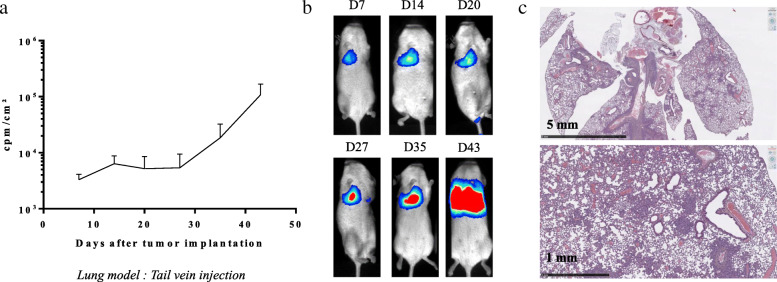
Development of the orthotopic PC-9 lung tumor model. PC-9 Luc^+^ cells were injected into the tail vein of NSG mice and **(a** & **b)** tumor growth was assessed by bioluminescence on days 7, 14, 20, 27, 35 and 43 after tumor cell injection. **a** Results show the evolution of the bioluminescence intensity over time and are expressed in cpm/cm^2^ for the thoracic area (mean ± SEM, *n =* 5). **b** Pictures show representative bioluminescence results of one PC-9 NSCLC Luc^+^-bearing mouse. **c** On day 43 after tumor cell injection, lungs from a PC-9 tumor-bearing mouse were collected and used for H&E staining. Pictures show representative results

In order to generate orthotopic tumors in the lung, PC-9 and A549 Luc^+^ NSCLC cells were injected intravenously, and tumor growth was assessed. Of note, the first assays were performed using Balb/c nude mice and Nod/Scid mice. In both of these cases, no tumor engraftment was observed by bioluminescence (*n* = 5, data not shown). Thus, injections were thereafter performed in NSG mice; NSG is one of the most highly immunodeficient mouse strains. As demonstrated by bioluminescence monitoring, intravenous injection of PC-9 Luc^+^ NSCLC tumor cells in NSG mice led to pulmonary implantation and tumor growth (Fig. 2a-c).

The lung tropism of tumor cells in this mouse model is consistent with the clinical observations made in patients with advanced disease. Indeed, H&E staining shows lung tumors characterized by scattered tumor cells (Fig. 2c). Tumor cells are organized in clusters or aligned along the alveolar walls. Of note, similar results were obtained with A549 Luc^+^ cells (data not shown).

**Fig. 3:**
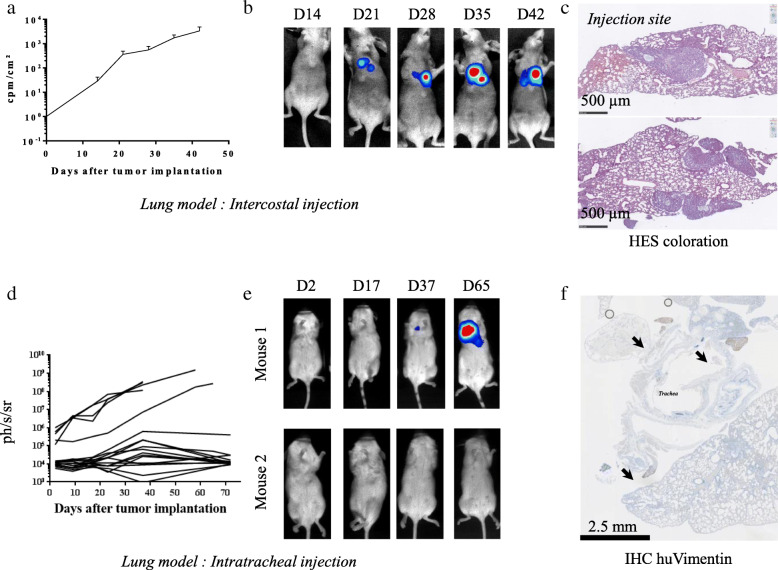
Evaluation of intercostal and intratracheal administration for setting up an orthotopic model of NSCLC tumors in mice. NSCLC Luc^+^ cells were injected by the intercostal (**a**, **b** and **c**) or intratracheal (**d**, **e** and **f**) route in immunodeficient mice. Tumor growth was assessed by bioluminescence. **a** & **d** Results show the evolution of bioluminescence intensity over time and are expressed in cpm/cm^2^ for the thoracic area (mean ± SEM, *n =* 5). **b** & **e** Pictures show representative bioluminescence results of NSCLC Luc^+^ tumor-bearing mice. **c** & **f** At a late stage in tumor development, lungs from PC-9 tumor-bearing mice were collected and used for H&E staining and vimentin IHC staining. Pictures show representative results. Black arrows show tumor nests

Simultaneously, we also assessed injections of NSCLC Luc^+^ cells by the intrathoracic and intratracheal routes (Fig. 3a-f). For the intrathoracic injection, Balb/c nude mice underwent A549 Luc^+^ injection and were monitored by bioluminescence (*n* = 6) (Fig. 3a-c). The results showed that the bioluminescence intensity increased over time after injection (Fig. 3a) and the localization of the signals suggests that the tumors were in the lungs for all of the mice (Fig. 3b). However, bioluminescence assays do not allow to distinguish whether the tumor cells are present in or around the lung. H&E staining performed on lung tissue sections 30 days after cell injection showed that, although a tumor mass was observed at the injection site (Fig. 3c upper picture), tumor development took place mostly around, but not inside the lung (Fig. 3c lower picture). For the intratracheal injection, the experiment was performed using Nod-Scid mice in order to favor tumor engraftment when compared to Balb/c nude mice. A549 Luc^+^ cells were injected intratracheally and monitored by bioluminescence (*n* = 17) (Fig. 3d-f). Results showed that the bioluminescence intensity increased over time after injection for only a few mice (*n* = 4/17) (Fig. 3d). For the few mice exhibiting tumor growth, bioluminescence signals also suggested that the tumor was localized in the lung (Fig. 3e). However, as observed with the intercostal injection, tumor development did not take place inside the lung, but mostly in the upper airways (larynx and bronchi) (Fig. 3f). Of note, these experiments were performed with A549 Luc^+^ NSCLC cells only. As true tumor development within the lung was not achieved, we decided not to perform the same experiments with the PC-9 cell line.

**Fig. 4:**
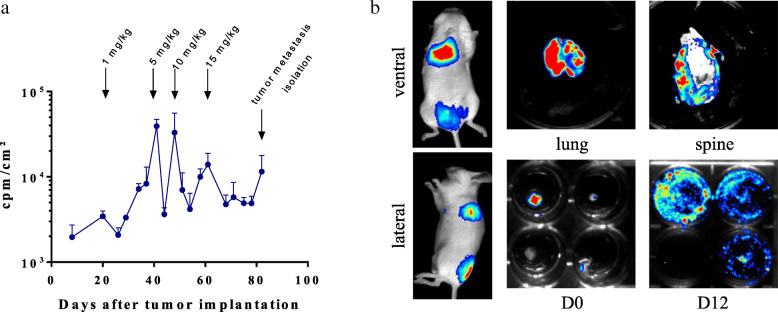
Osimertinib treatment of PC-9 Luc^+^ orthotopic tumor-bearing mice and tumor cell isolation from micro-metastases. PC-9 Luc^+^ cells were injected into the tail vein of NSG mice. Starting on day 21 after tumor cell injection and until the end of the experiment, mice were treated with increasing doses of osimertinib from 1 mg/kg to 15 mg/kg by ip injection 5 days per week. **a** PC-9 Luc^+^ tumor-bearing mice were evaluated by bioluminescence. Results show the evolution of bioluminescence intensity over time and are expressed in cpm/cm^2^ for the thoracic region (mean ± SEM, *n* = 3). On day 82 after tumor cell injection, the mice were euthanized, the organs that bore tumors were collected, and tumor cells were isolated to establish new cell lines. **b** Representative images of bioluminescence signals from PC-9 Luc^+^ tumor-bearing whole mice (top and bottom left images), lung and spine after tumor removal (top middle and right images) and backbone fragments cut soon after isolation (bottom middle image) and after being cultured for 12 days (bottom right image)

Osimertinib treatment was then evaluated using the NSG-mice orthotopic model after iv injection of PC-9 Luc^+^ tumor cells (*n* = 7). Tumor growth was monitored by bioluminescence on a regular basis after tumor cell implantation (Fig. 4a). In response to significant tumor growth, osimertinib was administered five times a week until the end of the experiment. When tumor rebound was observed, increasing doses of osimertinib were used (1, 5, 10 and 15 mg/kg starting on days 21, 41, 48 and 61, respectively). When tumor cells escaped the treatment, metastases were isolated (in this case, on day 82). Briefly, NSG mice were monitored for bioluminescence both ventrally and laterally to localize metastatic tumor sites (Fig. 4b, top and bottom left). Then, NSG mice were euthanized and the tumor sites were resected and analyzed for bioluminescence, including lung and bone metastases (Fig. 4b, top middle and right). As the tumor cells were not detectable in any way by macroscopic observations, these results demonstrated that the use of bioluminescence is absolutely required for early localization of tumor nests. Finally, bioluminescent tissue samples were cut into 4–5 mm^3^ fragments and placed in 24-well plates containing 10% FBS-RPMI in order to establish osimertinib-resistant cell lines. Bioluminescence analysis was performed shortly afterwards (Fig. 4b, bottom middle), and then several days later, when the tumor cell cultures began to grow steadily (Fig. 4b, bottom right). These established tumor cell lines may now be analyzed for osimertinib response and further characterized to investigate any resistance mechanisms.

## Discussion

NSCLC is the most common form of lung cancer and remains extremely deadly. Many new therapies for NSCLC are currently being assessed such as inhibitors targeting EGFR [19]. EGFR mutations are frequent in NSCLC; therefore, patients can be treated with EGFR TKIs. Currently, third generation inhibitors, such as osimertinib, are being evaluated in phase 3 clinical trials with encouraging results [20], and osimertinib has been approved by the FDA as a frontline treatment for patients with NSCLC who have tumors harboring EGFR mutations. Nevertheless, some resistance mechanisms are observed in response to these treatments, including third generation TKIs [13].

Some studies have aimed at setting up osimertinib-resistant cell lines in order to study these resistance mechanisms in vitro [21]. Others have developed ex vivo approaches, such as for brain metastases following implantation of tumor cells either directly into the brain [22] or into the left cardiac ventricle [23]. Our goal was to develop a relevant NSCLC mouse model that allows the isolation of tumor cells right at the beginning of tumor escape from osimertinib treatment. The model that we have developed makes it possible to follow the response to treatment, the relapse over a period of time which is long. It’s like following the natural course of the disease in a patient. This method allows further and deeper analyses, especially regarding early mechanisms implicated in tumor progression and relapse. Isolation of tumor cells, notably from bone micro-metastases, was possible using bioluminescence while these tumors were not yet detectable macroscopically.

The model developed herein is based on iv injection of PC-9 Luc^+^ cells into NSG mice. Several other NSCLC models have been developed, such as ectopic implantation of tumor cell lines and PDX (patient-derived xenografts). These sc models, suitable for longitudinal monitoring by direct measurements, are not representative of an original NSCLC tumor that starts in the lung. Another approach consists of using genetically engineered mouse models (GEM) [24]. These models are very powerful tools to analyze the early steps of oncogenesis. However, longitudinal monitoring of tumor growth is difficult to perform because of the lack of tools to detect and monitor tumor cell growth early.

With regards to orthotopic lung tumor models, the major explored routes consist of intrathoracic or intratracheal cell injection. Following intrathoracic administration, as previously demonstrated [25–31], NSCLC tumor cell development takes place at the injection site and also mainly around the lung, while deep invasion of tumor cells into the lung is rarely observed. Of note, Isobe and colleagues, who have shown tumor development inside the lung following surgical intercostal injection, have used small cell lung cancer (SCLC) cell lines and not NSCLC cell lines [28]. For intratracheal models [32, 33], tumor development may not deeply infiltrate healthy lung tissue. Interestingly, after intratracheal injection, tumors easily implanted in the upper airways, in a manner similar to a squamous cell carcinoma, which is often characterized by a proximal development, while a distal tumor localization is most frequently observed for human patients with a lung adenocarcinoma (original tumor type of PC-9 cell line) [34]. In this context, we have chosen to inject the tumor cells via the iv route, as previously described [35]. This injection route favors the localization and infiltration of PC-9 Luc^+^ tumor cells deep inside the lung, similar to what is observed in human NSCLC adenocarcinoma. Interestingly, we have previously performed the same experiments using other tumor cell lines, and they do not lead to the same features. For example, iv injection of H1650 Luc^+^ cells induces tumor development that is mainly localized within the liver (data not shown). These kinds of results and differences should be further studied.

The model presented here is based on the injection of human tumor cells, with the intention to study the mechanisms for relapses that can occur in human lung cancer after treatment. The same approach can also obviously be used in immunocompetent mice to study syngeneic tumors. Interestingly, our data show that, while injecting PC9 Luc^+^ cells leads to tumor engraftment after sc implantation in Balb/c mice, no tumor development was detected in Balb/c nude or Nod-Scid mice following iv injection. Only the highly immunocompromised NSG mice showed tumor development within the lung. Balb/c nude mice are deficient in T cell responses and Nod-Scid mice are deficient in B cell and T cell responses. NSG mice share Nod-Scid properties combined with IL-2Rγ deficiency, which disables several cytokine signaling pathways and results in a lack of functional NK cells. These data confirm, first, that the antitumor immune response is quite different depending on the tumor microenvironment, and, second, indicate that NK cells play an important role in lung immune surveillance [36] and are therefore an interesting target for immunotherapy [37].

In order to mimic current patient care, tumor-bearing mice were treated with osimertinib once the orthotopic lung tumor was established and its growth was sustained. As demonstrated by the Flaura clinical trial (NCT02296125) (PMID 29151359), osimertinib showed an efficacy superior to standard EGFR-TKIs in the first-line of treatment of *EGFR* mutation–positive advanced NSCLC, with a similar safety profile and lower rates of serious adverse events. However, NSCLC also frequently disseminates to the bones [3] and the efficacy of osimertinib against bone-metastatic EGFR-mutated NSCLC remain to be investigated. In this model, tumor regression was observed at first in response to osimertinib but, despite treating the mice with increasing doses, systematic regrowth was observed, associated with the development of bone metastases. Interestingly, bone micro-metastases were easily detected using bioluminescence, but were not possible to detect by macroscopic observation. Indeed, luciferase reporter gene detection is a fast and very sensitive technique for monitoring tumor growth, as well as the occurrence of micro-metastases, as it allows detection of only a few cells [38]. In-house experiments showed that we were able to detect as few as 500 PC-9 Luc^+^ cells after sc implantation (data not shown), a quantity of cells that is obviously impossible to observe by direct macroscopic approaches in living organisms.

Interestingly, after tumor escape (in this case, after treatment with a dose of 15 mg/kg osimertinib) micro-metastases could be isolated and transferred to culture dishes for the establishment of new tumor cell lines. This new tool will allow us to study the resistance mechanisms that occur at the beginning of tumor escape, and also to evaluate new alternative therapeutic strategies (currently studied in our laboratory).

## Conclusion

In conclusion, this model provides new biological tools to study tumor progression from the establishment of a lung tumor to the generation of drug-resistant micro-metastases, mimicking the natural course of the disease in human NSCLC patients, and to isolate appropriate cell lines derived from these metastases for further in vivo assays. The tumor cell lines thus generated will provide necessary access to a better understanding of tumor resistance mechanisms, such as those that take place after escape from osimertinib therapy. In addition, this orthotopic lung tumor model, in which tumor growth can be monitored by bioluminescence, is an innovative tool to evaluate the effectiveness of new therapies.

## Supplementary Information

**Additional file 1: Supplemental Data Table 1.**Summary of in vivo experiments.

## Data Availability

The datasets used and/or analyzed during the current study are available from the corresponding authors upon reasonable request.

## Abbreviations

BSA: Bovine Serum Albumin
DAB: Diaminobenzidine
EGFR: Epidermal Growth Factor Receptor
FBS: Fetal Bovine Serum
FELASA: Federation of European Laboratory Animal Science Association
GEM: Genetically Engineered Mouse model
H&E: Hematoxylin and Eosin
HRP: Horseradish Peroxidase
IHC: Immunohistochemistry
Luc +: Luciferase-positive
NSCLC: Non-Small Cell Lung Cancer
PBS: Phosphate-Buffered Saline
PDX: Patient-Derived Xenograft
PFA: Paraformaldehyde
SCLC: Small Cell Lung Cancer
SPF: Specific Pathogen-Free
TKI: Tyrosine Kinase Inhibitor
