# Correction to: Orthotopic model of lung cancer: isolation of bone micro-metastases after tumor escape from Osimertinib treatment

**DOI:** 10.1186/s12885-021-08339-w

**Published:** 2021-05-19

**Authors:** Ulrich Jarry, Mégane Bostoën, Raphaël Pineau, Laura Chaillot, Valentine Mennessier, Pierre Montagne, Emilie Motte, Marjorie Gournay, Arnaud Le Goff, Thierry Guillaudeux, Rémy Pedeux

**Affiliations:** 1 Université Rennes 1, UMS 3480 CNRS/US018 INSERM BIOSIT, Laboratoire Commun ONCOTRIAL, Rennes, France; 2 Biotrial Pharmacology, Unité De Pharmacologie Préclinique, Rennes, France; 3 INSERM U1242 COSS, Université Rennes 1, Clcc Eugène Marquis, Rennes, France

**Correction to: BMC Cancer 21, 530 (2021)**

**https://doi.org/10.1186/s12885-021-08205-9**

Following publication of the original article [1], the authors reported that the corresponding authors were incorrectly allocated:

The corresponding authors are Rémy Pedeux (remy.pedeux@univ-rennes1.fr) and Ulrich Jarry (ulrich.jarry@univ-rennes1.fr)

The original article [1] has been corrected.
